# How to Improve the Resolving Power of Compact Electrospray Ionization Ion Mobility Spectrometers

**DOI:** 10.1021/acs.analchem.3c00471

**Published:** 2023-05-16

**Authors:** Christian Thoben, Christian-Robert Raddatz, Aykut Tataroglu, Tim Kobelt, Stefan Zimmermann

**Affiliations:** Institute of Electrical Engineering and Measurement Technology, Department of Sensors and Measurement Technology, Leibniz University Hannover, Appelstraße 9A, 30167 Hannover, Germany

## Abstract

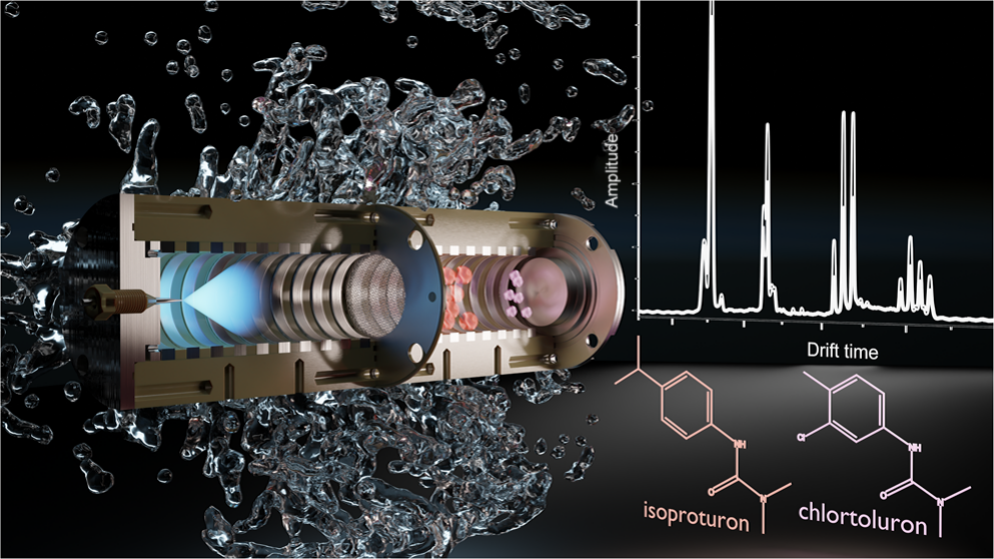

Every drift tube ion mobility spectrometer (IMS) has an optimum drift voltage to reach maximum resolving power. This optimum depends, among other things, on the temporal and spatial width of the injected ion packet and the pressure within the IMS. A reduction of the spatial width of the injected ion packet leads to improved resolving power, higher peak amplitudes when operating the IMS at optimum resolving power, and thus a better signal-to-noise ratio despite the reduced number of injected ions. Hereby, the performance of electrospray ionization (ESI)-IMS can be considerably improved. By setting the ion shutter opening time to just 5 μs and slightly increasing the pressure, a high resolving power *R*_P_ > 150 can be achieved with a given drift length of just 75 mm. At such high resolving power, even a mixture of the herbicides isoproturon and chlortoluron having similar ion mobility can be well separated despite short drift length.

## Introduction

Recently, ion mobility spectrometers (IMSs) have evolved from a detector for chemical warfare agents^1−3^ and explosives^4^ to a widely used instrument for analytical,^5−7^ medical,^8,9^ and bioanalytical applications.^10−13^ The accompanying increasing complexity of measurement tasks demands ever-higher analytical performance. Especially with electrospray ionization, high resolving power is required to separate, for example, protonated ions from their sodium adducts.^14−16^

An IMS separates ions based on their ion mobility in a neutral drift gas using an electric field. The drift velocity depends on the mobility and the applied electric field. Consequently, the strength of the drift field, or the total drift voltage applied to the drift tube, is one of the most important experimental parameters. Therefore, analytical models have been developed to understand its influence on the resolving power.^17−22^

The resolving power of an ion mobility spectrometer, defined as the ratio of drift time to peak width at half-height, can be described by eq 1(20,22)
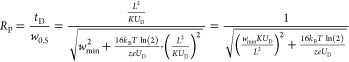
1with the length of the drift tube *L*, the drift voltage *U*_D_ applied, the minimum peak width *w*_min_ defined by the initial ion packet width and amplifier distortion, the mobility *K* of the ions and their charge state *z*, and the absolute temperature *T* as well as the Boltzmann constant *k*_B_ and the elementary charge *e*.

As can be seen from eq 1, resolving power is limited by the two terms of the square root: The left term considers the initial ion packet width and signal distortion caused by the amplifier giving the minimum peak width *w*_min_. In particular, for very narrow peak widths, the limited bandwidth of the amplifier electronics leads to peak broadening (signal distortion by the amplifier). The right term considers the peak broadening due to diffusion in the drift tube and depends on the temperature, the charge state, and the drift voltage. If we assume that mainly singly charged ions are generated with ESI, as is the case for smaller molecules, and consider the temperature as given, peak broadening by diffusion can only be reduced by the factor of the drift voltage. For the left term, since our analyte has a given reduced ion mobility *K*_0_ and we aim for compact IMS (and thus a reduced drift length, which is also beneficial looking at the high-voltage driver electronics), only the pressure, the initial ion packet width, and amplifier distortion as well as the drift voltage remain as variables. Equation 1 also reveals that the resolving power has an optimum with respect to the drift voltage since the term considering initial ion packet width and amplifier distortion increases with increasing drift voltage while the other term decreases with increasing drift voltage. The optimum drift voltage *U*_opt_ at maximum resolving power is given by eq 2, which can be determined from the derivative of eq 1.^22^ As seen from eq 2, the optimum drift voltage *U*_opt_ depends on the pressure *p*, the initial ion packet width, and the amplifier distortion giving the minimum peak width *w*_min_. At this optimum operating point, diffusion and the initial ion packet width and amplifier distortion equally contribute to peak broadening.
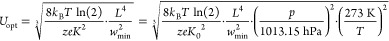
2Inserting eq 2 into eq 1 yields eq 3, describing the optimum resolving power *R*_opt_ obtained at *U*_opt_.^22^
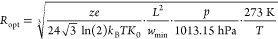
3For a given drift length, only minimizing the initial ion packet width and amplifier distortion or increasing the pressure will improve the resolving power. The effect of pressure on resolving power was initially investigated by Tabrizchi et al. using a corona discharge IMS in a lower pressure range between 39 and 776 hPa.^23^ Hill’s group later investigated the dependence of resolving power at pressures above 1013 hPa using a radioactive ionization source, omitting the variation of ion shutter opening time and thus initial ion packet width in his experiments.^24^ The IMS of Hill et al. has a drift length of 107 mm and achieves a resolving power around *R*_P_ = 60 at atmospheric pressure in a drift voltage range of 4–9.5 kV. With an increase in pressure to 2500 hPa, a resolving power close to *R*_P_ = 100 can be achieved with their setup. The above-mentioned work by Tabrizchi et al. achieves a resolving power around *R*_P_ = 50 at subatmospheric pressure, a drift length of 270 mm, and a drift voltage of 7.29 kV.

Other compact ESI-IMS achieve a resolving power of around *R*_P_ = 70, such as the system of Jafari et al. of 110 mm drift length and 6.6 kV drift voltage,^25^ or the commercial device of Exellims with a drift length of 105 mm and a drift voltage of about 4.9 kV.^26^ Our previous ESI-IMS setups at a pressure of around 1013 hPa, a drift length of 75 mm, and a drift voltage of about 5 kV already achieve resolving powers of *R*_P_ = 100.

However, minimizing the initial ion packet width in particular has a significant positive effect on the resolving power. Whereby the choice of the ion shutter is decisive in reducing the initial width of the ion packet. Only the ions that have completely passed the ion shutter at the end of the ion shutter opening time are actually injected into the drift tube since all other ions are discharged when the ion shutter closes.^27^ The distance that the ions must pass through the ion shutter region in order to be certainly injected is called the cut width. Since ions move with their characteristic drift velocity, the cut width causes the discrimination of ions with lower mobility.^28^ When the injection time is reduced, the same cut width leads to increasing discrimination of slow-moving ions, which is a major issue in ESI-IMS applications mainly focused on the analysis of larger ions.^29^

In particular, the above-mentioned ESI-IMS use Bradbury–Nielsen ion shutters with larger cut widths, while our design has practically no cut width since we use a tristate ion shutter allowing for short ion shutter opening times without any discrimination of slow-moving ions. For this reason, the proposed reduction of ion shutter opening times is not practicable with Bradbury–Nielsen ion shutters.^30,31^ Therefore, in the present work, we use a tristate ion shutter that does not show any ion suppression of less mobile ions^32,33^ and thus allows very short ion shutter opening times for the injection of the generated ions from the desolvation region into the drift region. In particular, larger ions with lower mobility are not discriminated by the tristate ion shutter, as has been shown for different IMS setups.^33−35^

## Experimental Section

### Instrumental

**Figure 1:**
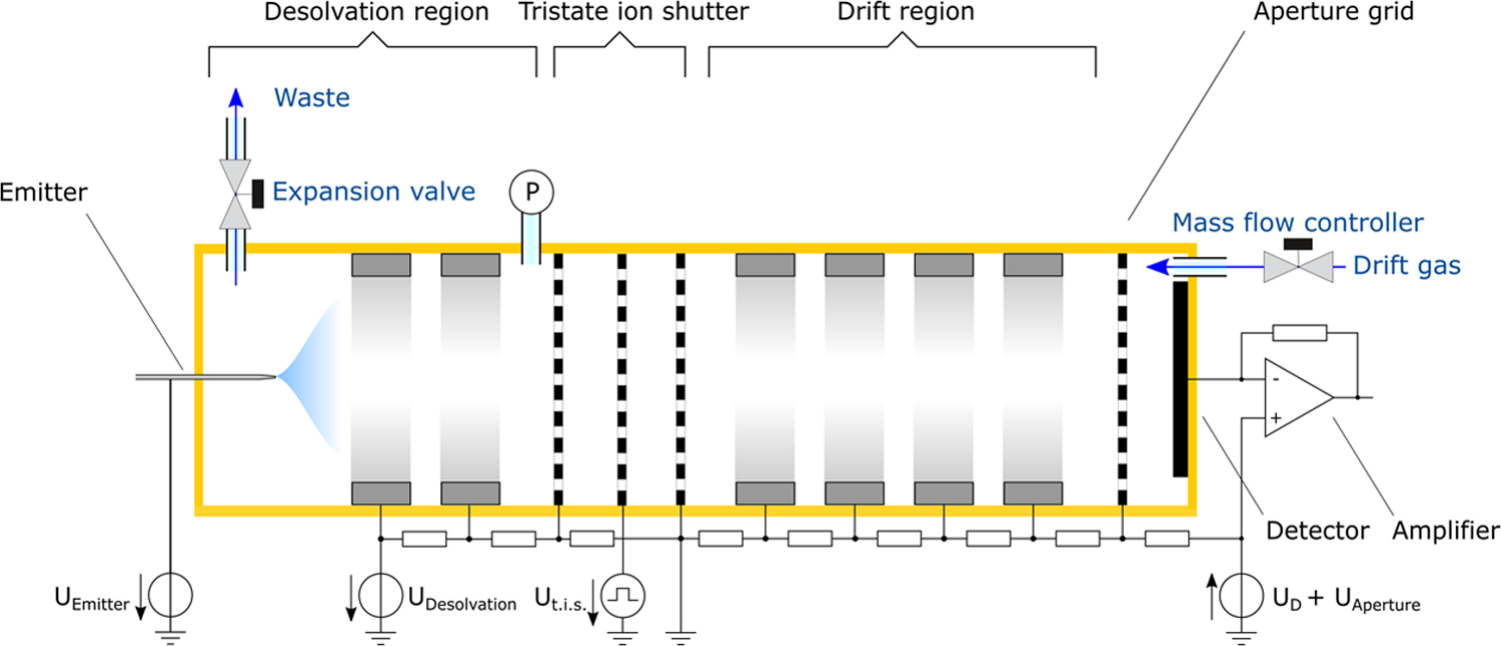
Schematic of the ESI-IMS.

In this work, a self-constructed compact ESI-IMS with a 75 mm drift tube length is used. A detailed description of the setup can be found elsewhere.^33^ The ions are generated by an electrospray ion source consisting of a metal emitter (New Objective Metal Taper Tip, DNU-MS, Berlin, Germany) with an inner tip diameter of 50 μm and a desolvation region of 50 mm length. The ion source operates at a flow rate of 2 μL/min. The ESI voltage of 2.7–3 kV is applied between the emitter and the first ring of the inlet of the desolvation region leading to an emitter needle-to-ring configuration. The ESI source is operated at room temperature without additional sheath, nebulization, or desolvation gas. The field strengths between the grids of the tristate ion shutter are adjusted to be twice the drift field strength. This results in an improved ion transmission through the ion shutter.^33^ The voltage pulse has a rise time of only 15 ns so that short opening times of 5 μs or even less can be realized.^32^ The third grid of the tristate ion shutter is pulled to ground. The voltage across the desolvation region is supplied by a 12.5 kV power supply from FuG (HCP35-12500). The drift voltage and the emitter voltage are powered by a 20 kV power supply from FuG (HCP35-20000). The pressure within the IMS is measured with a precision manometer from Greisinger electronic GmbH (GMH 3161-13) and adjusted via an expansion valve. Table 1 gives an overview of the relevant operating parameters of the ESI-IMS, and the setup is sketched in Figure 1.

**Table 1:** ESI-IMS Operating Parameters

parameter	value
length of drift region	75 mm
length of desolvation region	50 mm
drift field strength	65–227 V/mm
desolvation field strength	65–227 V/mm
emitter-to-ring voltage	2.7–3 kV
liquid flow to ESI	2 μL/min
emitter diameter	50 μm
drift gas flow rate	250 mL/min
drift gas dew point	–85 °C
drift gas	purified dry air
drift region temperature	25–29 °C
desolvation region temperature	25–29 °C
pressure (absolute)	800–1800 hPa

### Chemicals

LC-MS grade water and methanol (MeOH) were used as solvents and were purchased from Altmann Analytik GmbH & Co. KG, Germany. The herbicides isoproturon (analytical standard) and chlortoluron (analytical standard) as well as the instrument standard tetraoctylammonium bromide (TOAB) (ACS reagent) are analyzed in this work and were purchased from Sigma-Aldrich Chemie GmbH, Germany.

### Gas Supply

For use as drift gas, purified dry air with a dew point of −85 °C was supplied by a zero air generator (JAGZAG600S, JA-Gas Technology, Burgwedel, Germany) in combination with an air adsorption dryer K-MT 3 LAB (Parker Hannifin Manufacturing Germany GmbH & Co. KG, Essen, Germany) and with an additional moisture trap (Supelco, Molecular Sieve 5A Moisture Trap, 23991, Merck, Darmstadt, Germany) and an activated carbon filter (Supelcarb HC Hydrocarbon Trap, 24564, Merck).

## Results and Discussion

**Figure 2:**
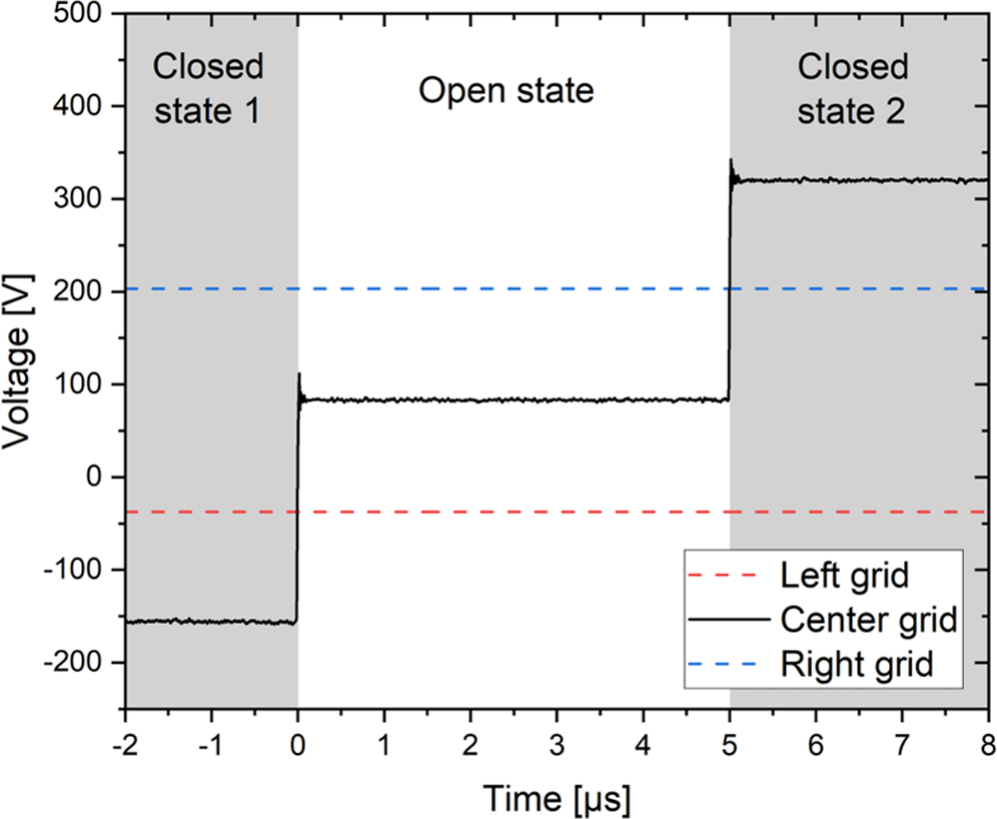
Measured voltage at the center grid of the tristate ion shutter (black solid line) over a sequence of the different states (−2 to 0 μs: closed state 1; 0–5 μs: open state; 5–8 μs: closed state 2) with the potentials of the left grid (red dashed line) and the right grid (blue dashed line).

To confirm the correct behavior of the voltages at the center grid of the tristate ion shutter even at very short open times of only 5 μs, we measured the voltage profile there. This is shown in Figure 2, where the two closed states of the ion shutter and the open state of 5 μs are clearly visible. The overshoot is due to the measurement setup used.

First, a sweep of the drift voltage is performed to experimentally determine the optimum drift voltage for the instrument standard tetraoctylammonium bromide (TOAB). In addition, the pressure within the IMS and the ion shutter opening time of the ion gate are varied systematically.

**Figure 3:**
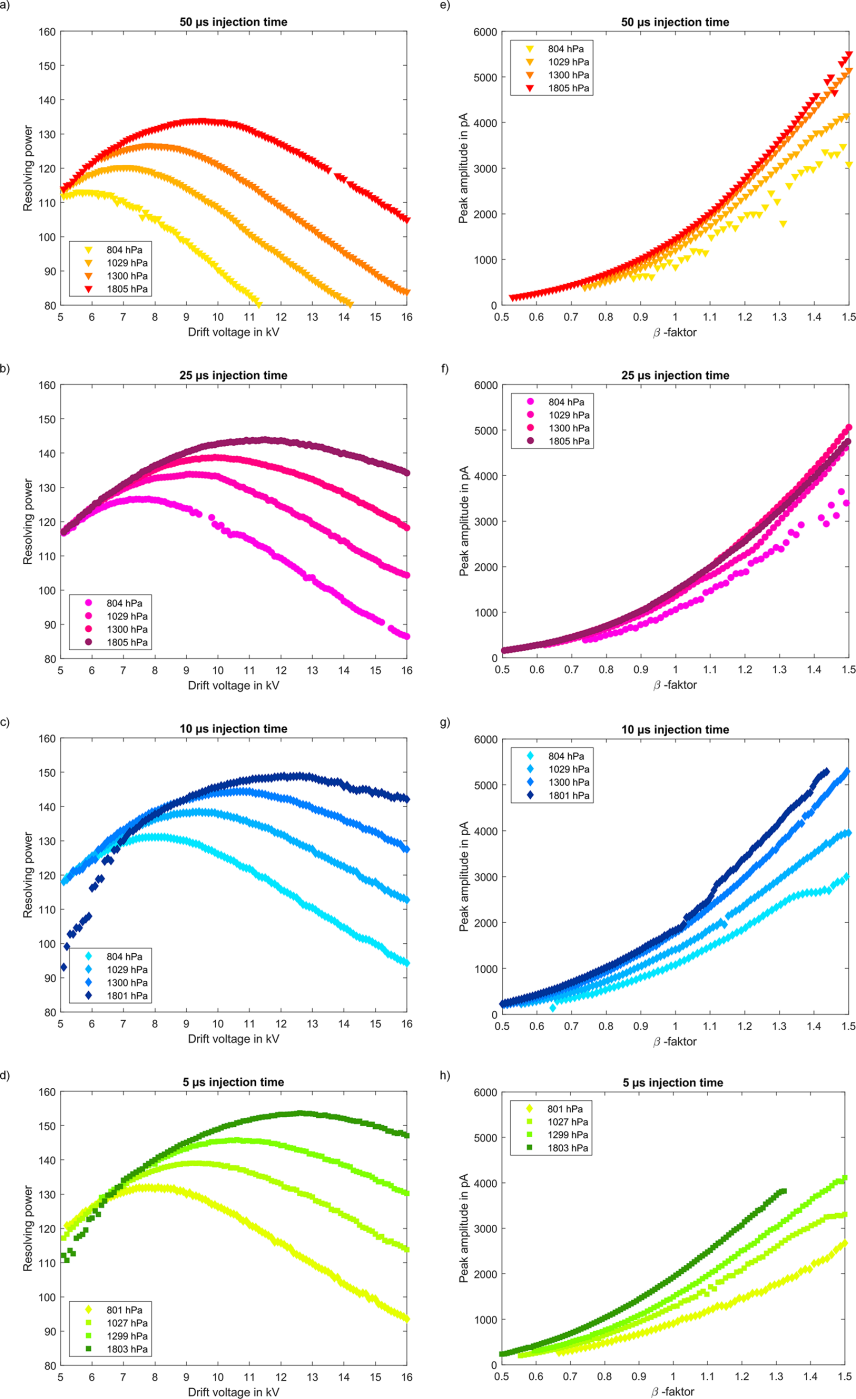
Resolving power (calculated from the TOAB peak present in the ion mobility spectra when electrospraying 100 μM TOAB in MeOH) versus drift voltage for ion shutter opening times of (a) 50 μs, (b) 25 μs, (c) 10 μs, and (d) 5 μs and peak amplitude versus β-factor for ion shutter opening times of e) 50 μs, f) 25 μs, g) 10 μs, and h) 5 μs as well as pressures of 801–804, 1027–1029, 1299–1300, and 1801–1805 hPa, respectively (ascending as color gradient).

Evidently, as described in eq 1, the resolving power increases for a given ion shutter opening time of 50 μs as the pressure within the IMS is increased, as shown in Figure 3. However, for maximum resolving power, higher and higher drift voltages are required with increasing pressure, which is also predicted by eq 2. It is also noticeable that above a certain drift voltage, the resolving power decreases again with further increasing the drift voltage. This follows from eq 1 since the first term in the square root dominates for high drift voltages and leads to a reduction in resolving power for higher drift voltages.

When the opening time of the ion gate is decreased, the same trend is seen when the pressure is increased. In addition, the optimum drift voltage for maximum resolving power shifts to higher drift voltages for shorter ion shutter opening times. At a pressure of 1803 hPa and an ion shutter opening time of just 5 μs, a high resolving power of *R*_P_ = 155 is achieved for the instrument standard at a given drift length of only 75 mm. Besides resolving power, another important parameter is the signal-to-noise ratio and thus the signal amplitude considering constant noise. In IMS, the signal amplitude can be increased by using a higher drift voltage than required for optimum resolving power. The ratio of the drift voltage to the optimum drift voltage for reaching optimum resolving power is the β-factor.^21^ Thus, the β-factor describes by what factor the drift voltage varies from the optimum drift voltage with respect to optimum resolving power. As shown in Figure 3, β-factor >1 gives higher signal amplitudes compared to the operating point for optimum resolving power. Since the optimal drift voltage for optimal resolving power is lower at longer injection times, a larger β-factor can be obtained for a given limited voltage supply. This is related to a larger increase in signal amplitude. Consequently, if the signal-to-noise ratio is to be optimized, resolving power must be foregone and longer injection times together with the highest possible drift voltage should be targeted. Furthermore, the signal amplitude at a given β-factor further increases with pressure. The reason for this is the required higher drift voltage at higher pressures to achieve the same β-factor as for low pressures. A similar behavior results when examining the optimal resolving power.

**Figure 4:**
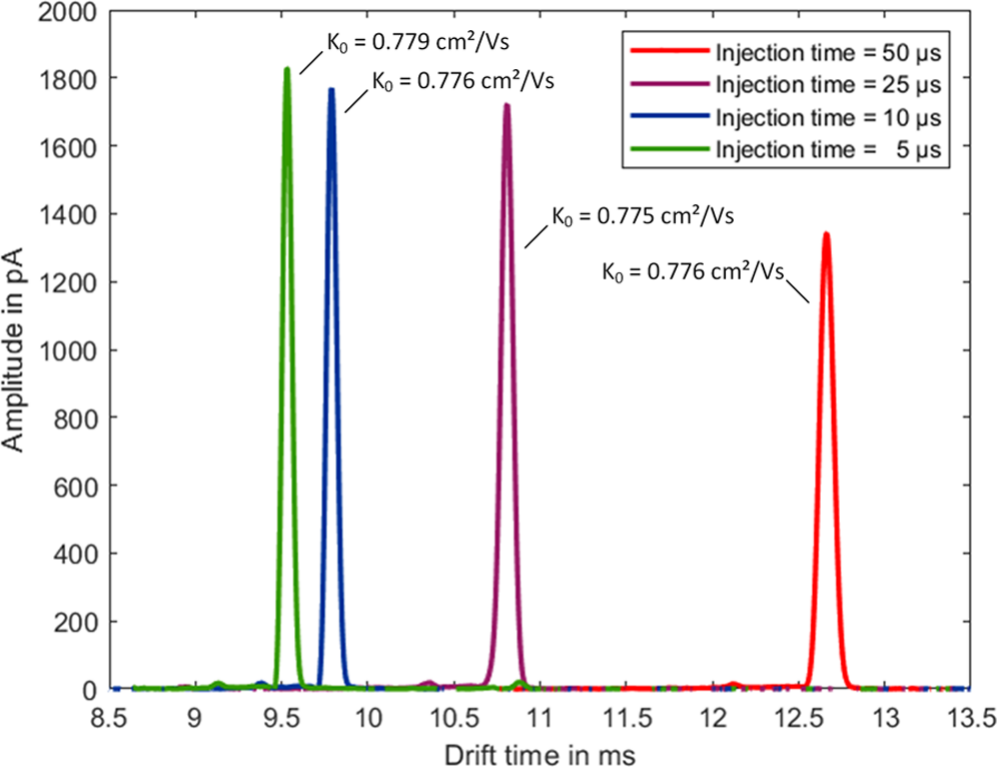
Ion mobility spectra (in each case, the TOAB monomer peak when electrospraying 100 μM TOAB in MeOH) recorded at the optimum drift voltages (9700, 11 400, 12 400, and 12 800 V, respectively) for maximum resolving power (*R*_P_ = 134, 144, 149, and 155, respectively) for the ion shutter opening times of 50 μs (red), 25 μs (purple), 10 μs (blue), and 5 μs (green) at a pressure of 1801–1805 hPa.

In Figure 4, the spectra at the respective optimum drift voltage for maximum resolving power at a pressure of 1801–1805 hPa are considered. For the injection time of 50 μs, the optimal drift voltage is 9700 V, resulting in a resolving power of *R*_P_ = 134. With the injection time of 25 μs, the optimal drift voltage is 11 400 V and the resolving power is *R*_P_ = 144. For the injection time of 10 μs, an optimal drift voltage of 12 400 V is necessary to achieve the resolving power of *R*_P_ = 149. And for the shortest tested injection time of 5 μs, the optimal drift voltage is 12 800 V, which leads to a resolving power of *R*_P_ = 155. It should be evident that improving the resolving power is not the same as optimizing the peak amplitude. However, looking at the spectra shown in Figure 4 recorded at the respective optimum drift voltage for maximum resolving power, it is noticeable that the peaks have shorter drift times due to the higher drift voltages required for maximum resolving power. But also, and even more important, the peak amplitudes increase with decreasing ion shutter opening times. This again shows that despite shorter ion shutter opening times and thus smaller numbers of injected ions, an increase in amplitude is possible. However, an increase of the ion shutter opening time at a given drift voltage leads to an increase of the signal amplitude but also to a reduction of the resolving power.

**Figure 5:**
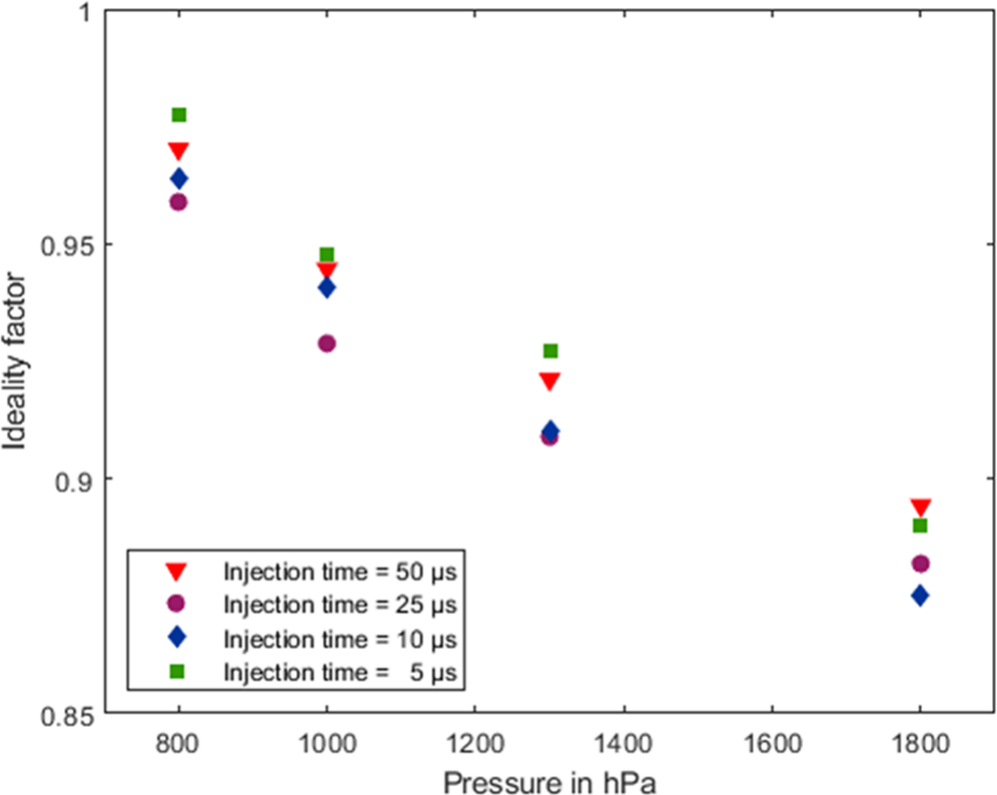
Ideality factor at different pressures within the IMS for each of the ion shutter opening times of 50 μs (red triangles), 25 μs (purple circles), 10 μs (blue diamonds), and 5 μs (green squares).

The so-called ideality factor describes how well the drift tube approaches an ideal drift tube, limited only by the unavoidable peak broadening defined by the minimum possible peak width (including the initial ion packet width and the amplifier distortion) and diffusion. It is consequently the ratio between the measured optimal resolving power and the theoretical optimal resolving power according to eq 3. The ideality factor can be calculated according to eq 4.^22^
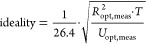
4Interestingly, the ideality factor increases as the pressure within the IMS decreases, as can be seen well in Figure 5. One possible explanation is that higher pressure shifts the IMS to an operating point that is more susceptible to nonidealities within the drift tube, such as field inhomogeneities.^36^ The influence of field inhomogeneities can be described by lengthening one of the trajectories by Δ*L* when ions of equal mobility *K* follow the field lines due to the drift voltage *E*_D_*L*; this leads to a relative peak broadening Δ*t*_D_/*w*_0.5_ as described by eq 5(18,20)
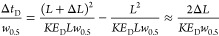
5At a higher pressure, the ion mobility decreases by a larger factor than the electric field must be increased to achieve the optimal resolving power. This leads to longer drift times and thus to a larger error due to field inhomogeneities. Therefore, for increased pressure, the ideality factor drops, or, in other words, at reduced pressure, the measured optimal resolving power is closer to the ideal, albeit lower, value.

**Figure 6:**
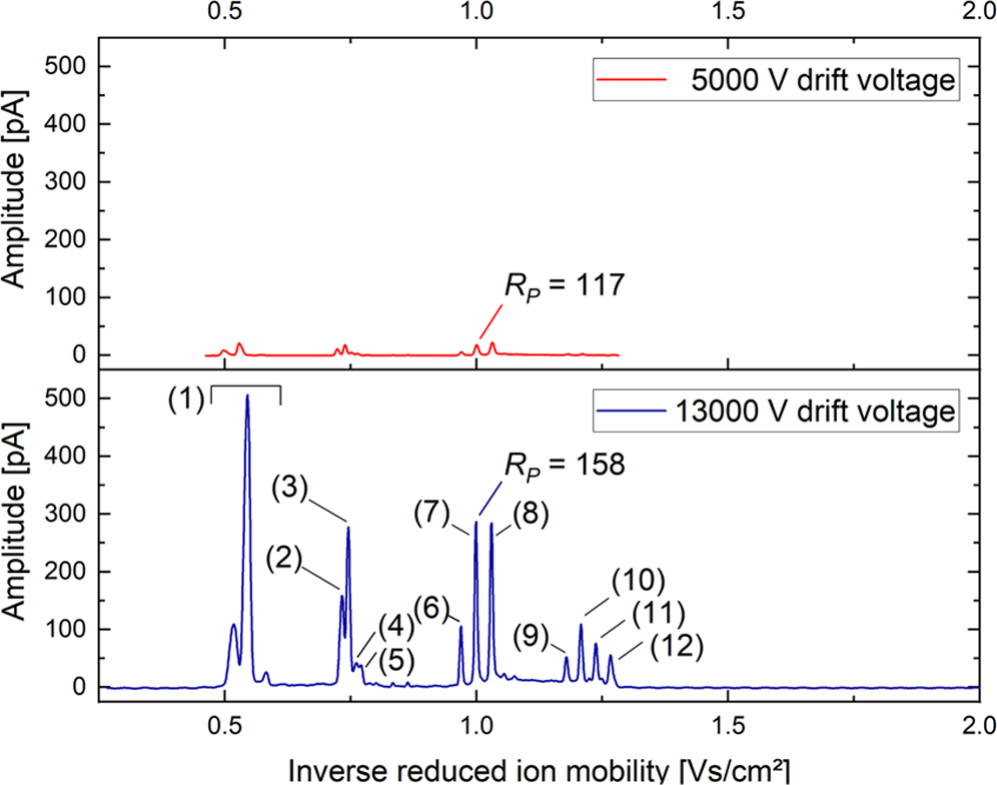
Ion mobility spectra of a mixture of 50 μM isoproturon and chlortoluron in 20:80 H_2_O/MeOH with an ion shutter opening time of 5 μs versus the inverse reduced ion mobility 1/*K*_0_ at 5000 V drift voltage (red) and 13 000 V drift voltage (blue) at a pressure of 1802 hPa. In addition to the solvent peaks (1), protonated isoproturon monomers (2) at *K*_0_ = 1.37 cm^2^/(Vs), protonated chlortoluron monomers (3) at *K*_0_ = 1.34 cm^2^/(Vs), sodium-bound isoproturon monomers (4) *K*_0_ = 1.31 cm^2^/(Vs), sodium-bound chlortoluron monomers (5) at *K*_0_ = 1.30 cm^2^/(Vs), protonated isoproturon dimers (6) at *K*_0_ = 1.03 cm^2^/(Vs), sodium-bound isoproturon dimers (7) at *K*_0_ = 1.00 cm^2^/(Vs), and mixed sodium-bound dimers of isoproturon and chlortoluron (8) at *K*_0_ = 0.97 cm^2^/(Vs), as well as trimers (9), (10), (11), and (12) at *K*_0_ = 0.85, 0.83, 0.81, and 0.79 cm^2^/(Vs), respectively, are detected.

Optimizing the resolving power of an IMS always has the objective of increasing the separation performance, especially if the IMS is to be used as a stand-alone device without pre-separation. Therefore, a worse ideality due to the increased pressure can be accepted for this purpose, as it still leads to an increase in resolving power. However, at the highest tested pressure of 1802 hPa and a short ion shutter opening time of 5 μs, very similar substances such as isoproturon and chlortoluron can be separated from each other due to a high resolving power of *R*_P_ = 155 reached at such high pressure and short ion shutter opening time. Figure 6 demonstrates the separation of isoproturon and chlortoluron including their dimers and trimers and their corresponding sodium adducts. The sodium to form the adducts may originate from the glassware, stainless steel, and tubing or may simply be an impurity in chemicals or solvents.^37−39^ The identification of the individual peaks was done using our ESI-IMS-MS coupling, which is described elsewhere.^40,41^ Since larger collision cross sections are documented for chlortoluron compared to isoproturon,^42^ it can be assumed in a first approximation that chlortoluron would show a lower ion mobility. However, the results from Figure 6 show the opposite; here, chlortoluron or its sodium adducts show higher ion mobility compared to isoproturon. In addition to the collision cross section, the charge and the mass, which in turn is larger for chlortoluron, also have an effect on the ion mobility. External factors such as pressure, temperature, and humidity in the drift gas also affect ion mobility. In addition, it is conceivable that in our IMS the analytes are still surrounded by a solvate shell, especially since the measurements were performed at room temperature. Conversely, the calculation of CCS values is often based on assumptions about the setup used, so these values are also subject to error. Despite the larger collision cross section, it is therefore plausible that chlortoluron exhibits a higher ion mobility compared to isoproturon. With 512 averages, the measurement at a pressure of 1802 hPa has a standard deviation in terms of drift time of 5 μs and amplitude of 10 pA.

Spectra were recorded at the optimum drift voltage of *U*_opt_ = 13 000 V for a maximum resolving power of *R*_P_ = 158 and at a much lower drift voltage of *U*_D_ = 5000 V used in our previous setup achieving a resolving power of *R*_P_ = 117 with otherwise identical operating parameters. For comparability, the spectra are plotted versus the inverse reduced ion mobility instead of the drift time. As expected, increasing the drift voltage results in a general increase of all peak amplitudes. For example, peak (7), which is the sodium-bound dimer of isoproturon, increases by a factor of 16. In addition, the resolving power of the considered peak increases from *R*_P_ = 117 to *R*_P_ = 158. The resolution, which describes the degree of separation between two peaks in terms of their average peak width at half-maximum, is defined as^43^
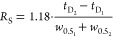
6As an example, between the peak (8) at *K*_0_ = 0.97 cm^2^/(Vs) (mixed sodium-bound dimer of isoproturon and chlortoluron) and the peak (7) at *K*_0_ = 1.00 cm^2^/(Vs) (sodium-bound dimer of isoproturon), the resolution increases from *R*_S_ = 2.0 to *R*_S_ = 2.8 at the optimum drift voltage of maximum resolving power. Thus, the peak capacitance for the presented ESI-IMS also increases.

However, the results shown here were obtained with a laboratory setup. In the future, the presented ESI-IMS will be further modified toward a portable, field-ready device. For this purpose, micromembrane pumps^44^ and small high-voltage DC/DC converters^45,46^ will be used to reach high pressures when needed and high voltage while keeping instrumentation portable.

## Conclusions

By using a tristate ion shutter, which allows very short ion shutter opening times of a few microseconds without any discrimination of large, slow-moving ions, specifically below a reduced ion mobility of *K*_0_ = 1.5 cm^2^/(Vs), the resolving power of an IMS can be significantly improved by increasing the drift voltage. It is possible to further increase the resolving power by increasing the pressure within the IMS, but this usually means a loss of ideality as field inhomogeneities have a greater impact. Reducing the ion shutter opening time while increasing the drift voltage is therefore the better way to increase the resolving power instead of increasing the pressure. In principle, these results are also applicable to IMS that ionize analytes in the gas phase, in particular when using longer reaction regions as for corona discharge ionization or Ni-63.

However, at a pressure of 1802 hPa and an ion shutter opening time of 5 μs, we achieve a resolving power of *R*_P_ = 155 for a given drift length of only 75 mm and when using the instrument standard tetraoctylammonium bromide. At such a high resolving power, even a mixture of the herbicides isoproturon and chlortoluron can be well separated.

Since the optimal drift voltage for the optimal resolving power is lower at longer injection times, a larger β-factor can be obtained for a given limited voltage supply so that higher signal amplitudes can be reached. Consequently, if the signal-to-noise ratio is to be optimized, resolving power must be foregone and longer injection times together with the highest possible drift voltage should be targeted.

With this improved compact, high-resolution ESI-IMS, more complex mixtures, e.g., in environmental or medical applications, will be analyzed in the future.
